# Flight Delay Regression Prediction Model Based on Att-Conv-LSTM

**DOI:** 10.3390/e25050770

**Published:** 2023-05-08

**Authors:** Jingyi Qu, Min Xiao, Liu Yang, Wenkai Xie

**Affiliations:** Tianjin Key Laboratory of Advanced Signal Processing, Civil Aviation University of China, Tianjin 300300, China

**Keywords:** flight delay prediction, deep learning, spatio-temporal characteristics, attention mechanism

## Abstract

Accurate prediction results can provide an excellent reference value for the prevention of large-scale flight delays. Most of the currently available regression prediction algorithms use a single time series network to extract features, with less consideration of the spatial dimensional information contained in the data. Aiming at the above problem, a flight delay prediction method based on Att-Conv-LSTM is proposed. First, in order to fully extract both temporal and spatial information contained in the dataset, the long short-term memory network is used for getting time characteristics, and a convolutional neural network is adopted for obtaining spatial features. Then, the attention mechanism module is added to improve the iteration efficiency of the network. Experimental results show that the prediction error of the Conv-LSTM model is reduced by 11.41 percent compared with the single LSTM, and the prediction error of the Att-Conv-LSTM model is reduced by 10.83 percent compared with the Conv-LSTM. It is proven that considering spatio-temporal characteristics can obtain more accurate prediction results in the flight delay problem, and the attention mechanism module can also effectively improve the model performance.

## 1. Introduction

For a long time, the basic contradiction in the development of civil aviation was that the supply capacity could hardly meet the fast-growing market demand, resulting in substantial fluctuations in flight punctuality and frequent flight delays. During the 13th Five-Year Plan period, the Civil Aviation Administration of China (CAAC) implemented the policy of controlling the total amount and adjusting the structure, the problem has been alleviated to a certain extent by artificially controlling the total number of flights [1]. From 2020, the total number of flights dropped sharply, and flight punctuality improved due to COVID-19 [2]. However, as the global epidemic situation slows down, the total number of flights will continue to rise, and flight delays in the post-epidemic era will continue to warrant attention [3]. Many factors can lead to flight delays, the most common ones include air traffic control reasons, weather reasons, airline reasons, and passenger reasons [4], and with the growth of flight volume, the proportion of irregular flights caused by weather is increasing, approaching 60% in 2021 [5]. Therefore, it is necessary to study the influence of weather factors on flight delays and improve the performance of flight delay prediction under bad weather.

The current flight delay prediction problem can be divided into two main categories: classification prediction of delay levels and regression prediction of delay times. Compared with classification prediction models, regression models can predict specific delay times, providing more granular guidance for practical application in the relevant sectors. The trending nature of the regression model itself makes it more advantageous in problems that examine significant associations between independent and dependent variables and the strength of the effects of multiple different independent variables on a dependent variable, and the flight delay problem is the result of the interaction of delay times with multiple characteristic factors in the data.

In the traditional machine algorithm to build the regression model, Luo et al. first used the phase space reconstruction theory to find that there are chaotic characteristics in the time series composed of flight arrival delays and built the flight delay prediction model by the support vector machine method [6]. Churchill et al. studied the delay spread of a single flight in multiple chain airports based on two machine learning models, logistic regression and decision tree. Through experimental comparison, the prediction error of the logistic regression model is lower [7]. Ma et al. established a chaotic short-term prediction model based on an extreme learning machine for the chaotic characteristics of flight delay time series [8]. Luo et al. first added the characteristics of the aviation information network to the airport data and used the SVR method to obtain a nonlinear regression prediction model [9]. He et al. built a flight delay prediction model based on the support vector machine regression method and used the feature with the highest correlation with the flight delay time in the data set as a variable to predict the overall delay level [10]. Feng et al. designed a web service based on the linear regression method to predict whether and how long a flight will be delayed [11]. Wang et al. built a flight delay model based on random forest regression and decision tree regression using real data sets from large domestic airports, and the model fit reached 0.83, reducing the risk of overfitting the model [12].

With the continuous development and progress of prediction algorithms and the successful application of deep learning methods in various fields, recurrent neural networks are especially widely used in regression problems with time series [13,14,15,16,17,18,19,20]. LSTM (Long Short-Term Memory) [21] has a strong feature extraction ability for time series data, so the prediction accuracy is significantly higher than traditional machine learning methods. Khanmohammadi et al. first adopted the model prediction of ANN, and the results were due to the traditional backpropagation algorithm [22]. Kim et al. construct sequences for flight data and, in a single airport, employ an RNN model to predict flight delays [23]. Li et al. built a regression prediction model based on the LSTM network by considering the correlation between the airline and airport in the time dimension and the space dimension which can fully extract the information of the flight data [24]. Fu et al. proposed a data augmentation method for the unbalanced characteristics of the flight delay data set which improved the prediction performance of the model to a certain extent [25]. Song et al. specifically constructed a neural network model for the flight segment from Shanghai Hongqiao Airport to Beijing Capital Airport to achieve dynamic prediction of flight arrival delays [26]. Wang et al. positioned their research on the departure delay time prediction of a single flight and dynamically updated the training data through the latest flight operation data to build a flight delay prediction model [27]. Zhang et al. collected the ADS-B signal as a dataset and extracted the spatial information in it and built a flight delay prediction model through the LSTM algorithm [28]. Chen et al. used the Conv-LSTM algorithm to extract both temporal and spatial features and verified the effectiveness of the model on an urban rail transit dataset [29]. Zhang et al. captured dynamic spatial dependencies through the PageRank algorithm, then input LSTM to weight the spatial dependencies, and finally added a temporal attention mechanism and introduced auxiliary features to improve the accuracy of the prediction model [30].

In the problem of flight delay regression prediction, a large number of scholars have studied the time dimension, but there are fewer flight delay prediction models that incorporate a comprehensive consideration of the spatial dimension. Aiming at the above problems, a flight delay prediction model based on Att-Conv-LSTM is proposed. On the basis of the time series in the extracted data, the meteorological data is added to expand the feature column, and the spatial information of the data is synchronously extracted by a convolution operation. It makes full use of the hidden spatial features within the samples and the temporal features between samples and then adds the attention mechanism module to improve the learning efficiency of the algorithm. In this paper, the validity of the proposed model is verified against the flight data of four domestic airports, and the influence of different factors, such as meteorological data and sequence length on the delay state, as well as the weight distribution of the attention module in the time series network are discussed.

## 2. Flight Delay Regression Prediction Model

**Figure 1:**
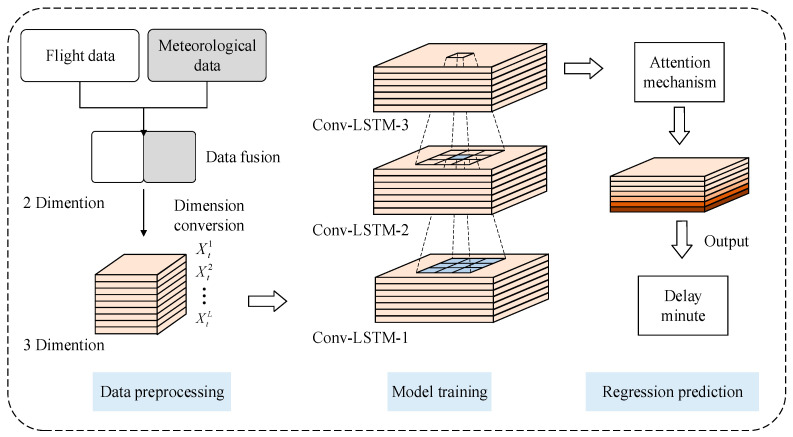
The overall structure of the flight delay regression prediction model.

The flight delay regression prediction model consists of three parts which are the data preprocessing when inputting the model, the training of the network, and the prediction when outputting the model. The entire prediction structure is shown in Figure 1. The preprocessing module mainly fuses flight and meteorological data, encodes the fused data and converts dimensions; the model training module is responsible for building the network, learning and training network parameters and saving the model; the prediction module directly outputs the predicted specific time of each flight delay.

### 2.1. Model Input

In a deep learning algorithm, the data set input to the model is a crucial part, and a high-quality data set can greatly improve the performance of the model. Before the original data is input into the neural network for training, data preprocessing is required first. The preprocessing process consists of four parts: data cleaning, data fusion, data encoding, and serialization. After data processing, all variables will be converted into numerical variables and normalized uniformly, and then the time series will be divided by a sliding window so that the samples have time series characteristics at the same time.

#### 2.1.1. Dataset Introduction

**Figure 2:**
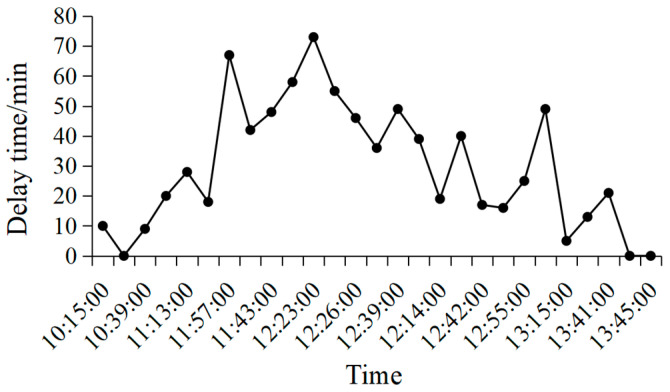
Statistics of flight delays at Tianjin Airport during a certain period of time.

The dataset used in this paper is the flight and meteorological data of four domestic airports provided by the North China Air Traffic Administration, namely Beijing Capital Airport, Beijing Daxing Airport, Tianjin Binhai Airport, and Shijiazhuang Zhengding Airport, with a sample size of 3.05 million, 790,000, 1.07 million, and 590,000, and the data was recorded from September 2019 to October 2020. The flight data includes 23 features, such as “flight number”, “aircraft model”, “take-off and landing time”, “take-off and landing airport”, “altitude”, and “speed”. Meteorological data is recorded every minute and includes 49 features, such as “runway visual range”, “rainfall”, “temperature”, and “wind speed”. In order to better describe the impact of flight delays on the time dimension, Figure 2 shows a statistical graph of flight delay time at Tianjin Airport from 10:00–14:00 on 1 October 2019.

The figure records the delay time of 27 flights during this period. It can be seen from Figure 2 that when the first flight is delayed, it will have a greater impact on the delay of several flights after a short period of time. With the passage of time, the delay time of subsequent flights gradually decreases which shows that the impact of flight delays only exists in several adjacent flights, and the number of flights affected by delays involves the choice of the parameter of time sequence in the network model.

#### 2.1.2. Data Preprocessing Process

**Figure 3:**
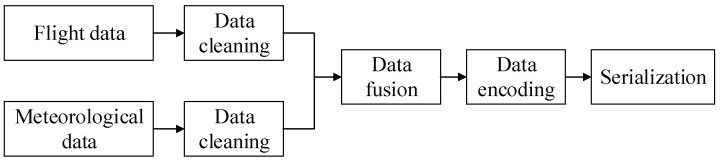
Data pre-processing diagram.

With the development of deep learning in recent years, the architecture of “code-neural network” has gradually matured. Therefore, keeping the architecture of the neural network fixed, focusing on the data, and improving the quality of the dataset is a more efficient way to improve the performance of the model. High-quality datasets can enable models to train and learn features more efficiently, so dataset preprocessing is particularly important. In order to obtain a high-quality dataset and make the data more suitable for the input of the network model, the original data needs to be cleaned first. After merging the cleaned flight data and meteorological data according to time, encode it, and finally construct time series samples through the sliding window and input them into the neural network model. The schematic diagram of the entire data preprocessing process is shown in Figure 3.

1. Data cleaning and labeling:

When the key features are missing in the data, the method used in this paper is to delete the entire flight or weather data; when the data contains outliers, the average value of the features is used to fill in. Since the neural network chosen in this paper is a supervised algorithm, after cleaning the data, we need to label the training set data.

2. Data fusion:

Putting the features of the data into the model completely and diversely helps the neural network to extract the information between the features. This paper fuses flight data and meteorological data, expands the feature columns of the dataset, and enriches the diversity of samples. In addition, the fusion of meteorological data will greatly increase the sample size and play a role in data enhancement, making the data set more suitable for the network model. Before fusion, it is necessary to extract the associated primary key as the basis for fusion. First, the planned departure time of the flight data is extracted, then the recorded time of the weather is extracted, and the two times are used as the key value for fusion; that is, when the two times are the same, combine the two flight and weather data into one. In this way, a piece of flight data with the weather information at the moment when the flight is scheduled to take off is obtained.

3. Data encoding:

In order to eliminate the influence between the dimensions of different feature columns, the data input to the neural network often needs to encode the data set into the same dimension and perform normalization processing, so that the neural network can better learn the association between the data. Since the fused data contains numerical features and discrete features, this paper adopts different coding methods for these two features [31]. The numerical features use Min-Max normalization coding, and the discrete features use Catboost coding [32].

**Figure 4:**
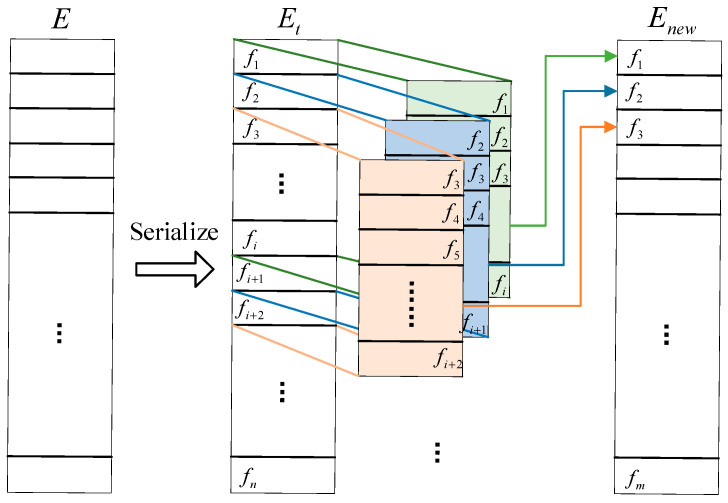
Serialization process.

4. Serialization:

Due to the short-term temporal correlation between flight delay data, we construct the input data as a time series, and the serialization process is shown in Figure 4. First, sort the data set according to the recording time to obtain the data set, then use the sliding window of the sequence length to perform sliding segmentation, slide down one data at a time, and obtain a new time series data of length. Use the label of the last flight data in the sequence data as the output of the network model.

After constructing the time series samples, it is necessary to divide the data set of the samples. According to general experience, the proportion of the sample size of the training set and the verification set in this paper is 8:2.

### 2.2. Model Output

The prediction of the regression model is the delay in outputting the prediction given the input variables. After data preprocessing, the input of the model is converted into tensors to fit the input dimension of the network. Table 1 lists the parameter structure of the input and output of the Att-Conv-LSTM network. The initial input dimension is (15, 72), that is, 15 pieces of data obtained after serialization are used as 1 sample, and each sample contains 72 columns of features. The construct of the Att-Conv-LSTM model makes its input require four-dimensional data, so the initial data are dimensionally transformed before being input into the model using the Reshape layer. After dimension conversion, a four-dimensional array of (15, 8, 9, 1) is obtained, where the last dimension is the number of filters. The output of each layer of the network is shown in Table 1. The output dimension indicates the dimension of the data after the current network layer. The parameter quantity represents the sum of the parameters in the current network layer and is directly related to the amount of space used in the disk for that layer of the network.

Since the regression model can obtain the specific delay time, it does not refer to the standard of no delay within 15 min stipulated by the Civil Aviation Administration of China. In order to evaluate the error of the model in the flight delay prediction problem, the evaluation standard used in this paper is *RMSE* (Root Mean Square Error), and its calculation formula is

(1) RMSE=(1n∑i=1n(yi−y⌢i)2)12,

where $y_{i}$ ${\overset{⌢}{y}}_{i}$ is the predicted value during training, and the calculation result is in minutes after the square and square root.

## 3. Conv-LSTM Network Based on Attention Mechanism

The attention mechanism was first applied to computer vision. In 2014, the Google team [33] added the attention mechanism to the deep learning recurrent neural network and achieved remarkable results in the problem of image classification. Then, the attention mechanism began to be widely used by scholars. Bahdanau et al. [34] applied it to the field of natural language processing and also obtained good results in translation algorithms. In 2017, the Google team proposed the Transformer encoder–decoder algorithm [35] which completely adopted the self-attention mechanism, abandoned the recurrent and convolutional neural networks commonly used in deep learning, and fully tapped the basic depth. The characteristics of neural networks are outstanding in many natural language processing tasks.

### 3.1. Network Description

**Figure 5:**
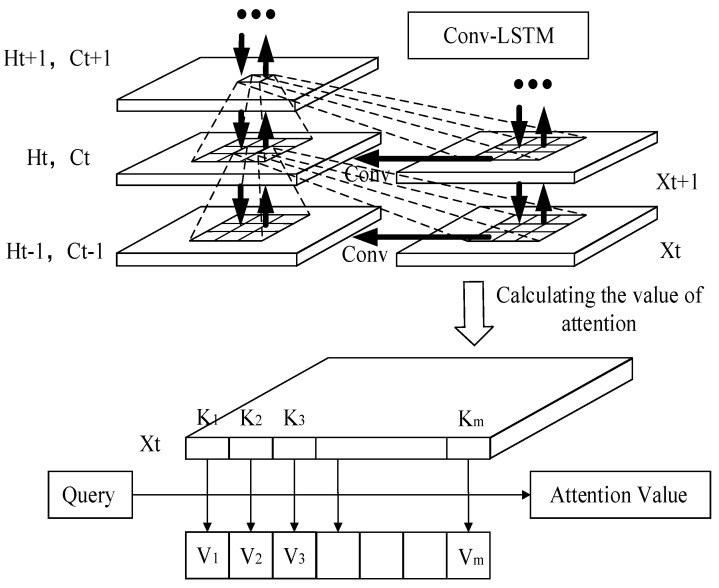
The network structure diagram of adding attention mechanism.

The essence of the attention mechanism stems from visual attention: when the visual system is facing a scene, it does not browse all the things in the scene but only looks at the places it focuses on. That is to say when the algorithm learns that in a scene, a certain part of the information is always highly related to the label; the next time it learns in a similar scene, the algorithm will focus on this information and try not to look at other sections for efficiency. The attention-based Conv-LSTM network structure is shown in Figure 5.

In the network structure, Query (hereinafter referred to as Q) is an element in a given target, and $K_{m}$ (hereinafter referred to as K) is a part of the key value that constitutes the element. That is, the Keywords: By calculating the relationship between Q and each K, you can get each weight coefficient of the corresponding value of K and then the weighted summation, the final attention weight value is obtained. The calculation steps can generally be divided into the following three steps: (1) Calculate the similarity between Q and K to obtain the weight; (2) Normalize the calculated weight; (3) With the normalized weights and $V_{m}$ weighted sum, the result of the weighted sum is the final attention value. However, since attention is not an independent model, it just adds new information, and its variant does not propose a new definition of network layer, so it can only be called an attention mechanism module not a new model.

The core part of the attention is a series of weight parameters. It iteratively learns the degree of association between each element and the label in the sequence and then reassigns the original input according to its correlation. The weight parameter is assigned by the attention module. The introduction of the attention mechanism module will assign different attention sizes to different vectors in a sequence, reflecting the influence of each vector on predicting the current information. Due to the introduction of new information, the efficiency of network learning will be greatly improved.

### 3.2. Feature Extraction

**Figure 6:**
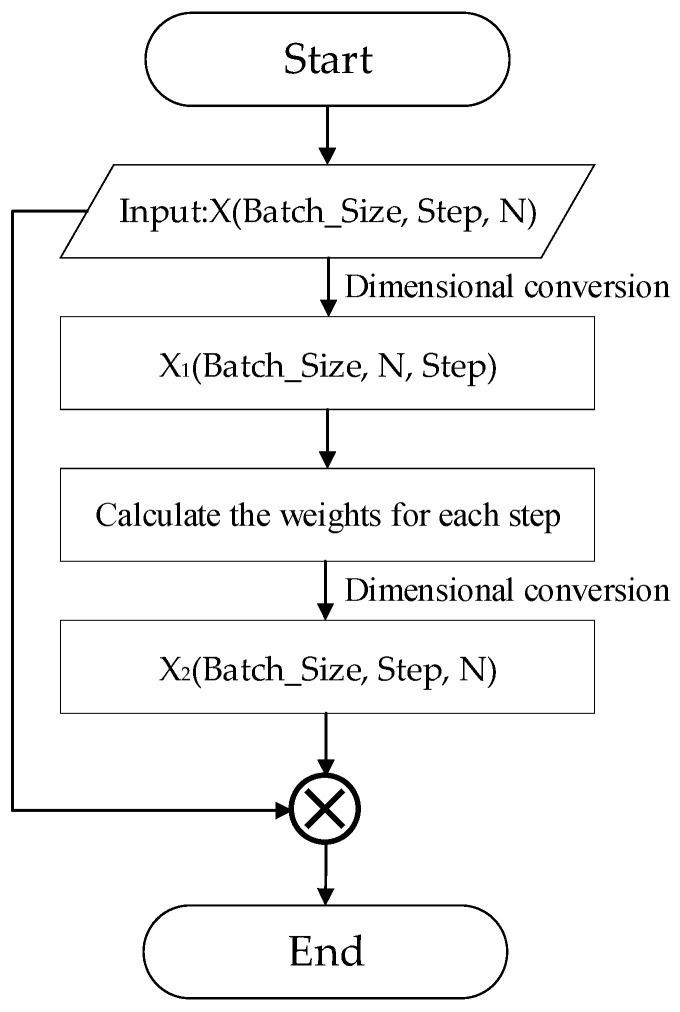
Schematic diagram of the attention mechanism.

The essence of the attention mechanism is to perform a series of weighted sum operations on the input by generating a weight coefficient for a specific label to identify the importance of the features in the input to the target. The following mainly introduces the basic principles of the attention mechanism to further understand the details of feature extraction inside the regression model. Its implementation is shown in Figure 6:

After the output of the Conv-LSTM network, we can obtain an output X with dimension (Batch_Size, Step, N), where Batch_Size is the size of the batch, Step denotes the length of the sequence, and N is the number of network cells. Firstly, X is treated as the feature of each time node as the input in Figure 6 which is transformed into X_1_ (Batch_Size, N, Step) after dimensional conversion to flip the second and third dimensions. Then, the weights of each feature in each step are calculated using the fully connected layer and Softmax classifier, and then the second and third dimensions are reduced after dimensional conversion to obtain X_2_ (Batch_Size, Step, N). Finally, X_2_ is multiplied with the input, that is, the weights of each step, multiplied by their features to obtain the final output value of the attention mechanism.

In order to realize the attention mechanism, we regard the input raw data as the form of <Key, Value> key-value pairs, and calculate the similarity coefficient between the keyword and the value according to the Query value in the given task target, and the corresponding value can be obtained. Then, use the weight coefficient to weight and sum the values to get the output. We use Q, K, and V to denote Query, Key, and Value, respectively. The formula for the attention weight coefficient W is as follows:

(2) $$W=soft\max(QK^{T})$$

**Figure 7:**
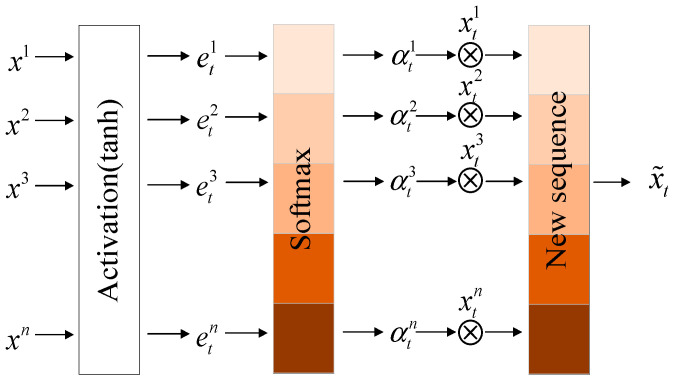
The principle of attention mechanism.

Figure 7 shows the input and output principles of the attention mechanism:

Taking a sample $x^{1},\ldots ,x^{n}$ with a sequence length of n as an example, as shown in Figure 7, after the output of the Conv-LSTM network, the attention mechanism module connected to it first assigns the sequence an tanh activation function to obtain the sequence ${e_{t}}^{1},\ldots ,{e_{t}}^{n}$, where $h_{t-1}$ represents the intermediate state, $C_{t-1}$ represents the hidden state, and U helps to find the optimal value during the iterative process. Then, through the softmax classifier, each vector in the sequence is assigned a weight value to obtain the weight sequence ${\alpha_{t}}^{1},\ldots ,{\alpha_{t}}^{n}$. Finally, the weight sequence is transposed and summed with the original input sequence to obtain the final output of the attention module. The calculation formula of the whole process is as follows:

(3) $${e_{t}}^{k}=V\tanh(W[h_{t-1},C_{t-1}]+Ux^{k}),$$

(4) $${\alpha_{t}}^{k}=\frac{exp({e_{t}}^{k})}{\sum_{i=1}^{n}exp({e_{t}}^{i})},$$

(5) $${\tilde{x}}_{t}={\left({\alpha_{t}}^{1}{x_{t}}^{1},\ldots ,{\alpha_{t}}^{n}{x_{t}}^{n}\right)}^{T}.$$

### 3.3. Model Training and Optimization

**Figure 8:**
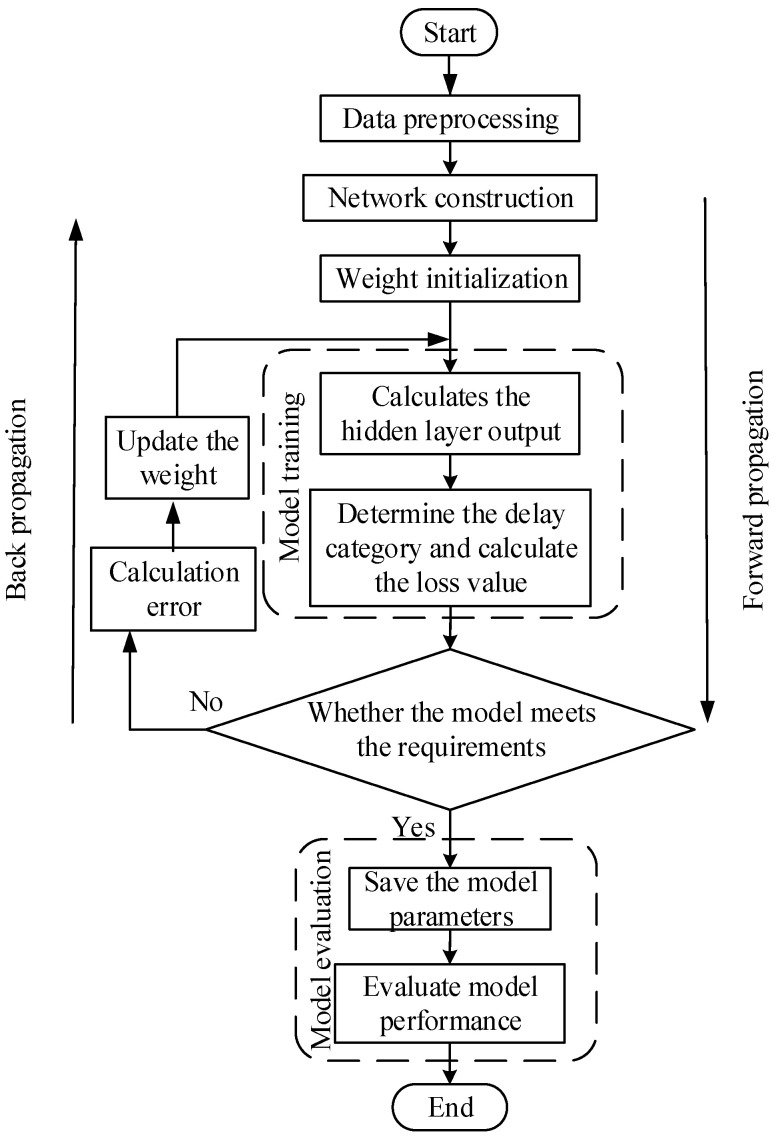
Flight delay regression model training flow chart.

The training iteration based on the Att-Conv-LSTM prediction model consists of forward propagation and back propagation which, respectively, completes the forward propagation from the shallow layer to the deep layer and the reverse back propagation for continuous error correction. The overall training process of the model is shown in Figure 8.

#### 3.3.1. Model Training

The training of the model consists of two parts, forward propagation and back propagation. In forward propagation, this paper defines the initial weight value, activation function, and error function. After forward propagation, the calculation result and the error value are obtained. In back propagation, the error of the output layer is input to the hidden layer through back propagation, the hidden layer adjusts the weight value, and performs forward propagation again, thereby performing an iterative process of network training.

In this paper, the BP [36] chain rule is used in the network training process to calculate the error term of the hidden layer, and then the weight gradient is calculated according to the error term. Formula (6) represents the derivation process for the weight matrix according to the full differentiation rule $\frac{\partial Z^{{\left(l\right)}^{T}}}{\partial W^{\left(l\right)}}=A^{\left(l-1\right)}$, where $Z^{\left(l\right)}$ denotes the state of the neuron at layer l and $A^{\left(l-1\right)}$ denotes the output of the neuron at layer l in matrix form. Then, calculate $\frac{\partial J}{\partial W^{\left(l\right)}}=\frac{\partial Z^{{\left(l\right)}^{T}}}{\partial W^{\left(l\right)}}⊗\frac{\partial J}{\partial Z^{\left(l\right)}}=A^{\left(l-1\right)}⊗\frac{\partial J}{\partial Z^{\left(l\right)}}$ according to the chain derivative rule, and write $\frac{\partial J}{\partial Z^{\left(l\right)}}$ as $\delta^{\left(l\right)}$ for convenience of representation. The momentum factor is updated according to the results of the weight derivation as shown in Formula (7), and finally, the weight matrix is updated by Formula (8) for the next iterative process. The calculation formula of the weight gradient is as follows.

(6) $$\frac{\partial J}{\partial W^{\left(I\right)}}=\delta^{\left(l\right)}⊗(A^{\left(l-1\right)}),$$

(7) $$V(t+1)=\mu V(t)-\eta [(\frac{\partial J}{\partial W(t)})+\lambda W(t)],$$

(8) W(t+1)=W(t)+V(t+1).

In the above formula, t is the number of iterations, μ is the momentum factor which indicates the influence of the correction range of the previous weight on the current weight value, V(t) is the momentum variable, and η is the learning rate which determines the speed of model training; λ is the weight decay coefficient.

In addition, deep learning generally divides the data set into training set and validation set. Each round of training will output the average loss value of the entire dataset through the above-mentioned forward propagation, and whether the loss value of training and validation is reduced synchronously as the standard, then continuously adjust the parameters in the network model through back propagation, and finally, obtain a set of parameters suitable for the network structure.

#### 3.3.2. Model Optimization

In this paper, the Adam [37] algorithm is used to optimize the network. This method is an improved first-order optimization algorithm based on the traditional stochastic gradient descent method which can dynamically change the weight value of the neural network during the iterative training of the neural network. The advantages of the Adam algorithm are: (1) The implementation method is simple, and there is no need to adjust hyperparameters. (2) It has an initial learning rate and can be adjusted automatically. (3) It is suitable for large sample size data and requires less memory. The detailed process of applying the Adam algorithm to gradient descent during neural network training is as follows: (1) Update the current number of iterations; as shown in Formula (9), t is the number of steps to update. (2) Calculate the gradient value of the network objective function to the parameters; as shown in Formula (10), θ is the parameter to be updated, f(θ) is the loss function, $g_{t}$ is the gradient obtained by the derivation of the objective function f(θ) to θ. (3) Calculate the first-order matrix of the gradient values; as shown in Formula (11), $\beta_{1}$ is the first-order moment decay coefficient, $m_{t}$ is the first moment of the gradient $g_{t}$. (4) Calculate the second-order matrix of the gradient; as shown in Formula (12), $\beta_{2}$ is the second-order moment Attenuation coefficient, $v_{t}$ is the second moment of the gradient $g_{t}$. (5) Correct the first-order matrix; as shown in Formula (13), ${\overset{⌢}{m}}_{t}$ is the bias correction of $m_{t}$. (6) Correct the second-order matrix; as shown in Formula (14), ${\overset{⌢}{v}}_{t}$ is the bias correction of $v_{t}$. (7) Update the parameter $\theta_{t}$ [38].

(9) t=t+1,

(10) $$g_{t}=\nabla_{\theta }f_{t}(\theta_{t-1}),$$

(11) $$m_{t}=\beta_{1}\cdot m_{t-1}+(1-\beta_{1})\cdot g_{t},$$

(12) $$v_{t}=\beta_{2}\cdot v_{t-1}+(1-\beta_{2})\cdot {g_{t}}^{2},$$

(13) $${\overset{⌢}{m}}_{t}=m_{t}/(1-{\beta_{1}}^{t}),$$

(14) $${\overset{⌢}{v}}_{t}=v_{t}/(1-{\beta_{2}}^{t}),$$

(15) $$\theta_{t}=\theta_{t-1}-\alpha \cdot {\overset{⌢}{m}}_{t}/(\sqrt{{\overset{⌢}{v}}_{t}}+\varepsilon ).$$

## 4. Results

This section will introduce the experimental environment and basic parameters and compare and verify the model performance of the algorithm through various indicators. The regression prediction model based on Att-Conv-LSTM is an improved model based on the Conv-LSTM algorithm. Therefore, it is necessary to compare the network performance of the two from various indicators, such as error, and analyze the influence of flight information, weather factors, and sequence length on flight delays. The effect of prediction is verified, and the effectiveness of attention is verified by comparing the experimental results of different prediction models.

### 4.1. Experimental Environment and Parameter Configuration

The experimental environment processor is Intel Xeon E5Mu1620, the GPU memory is 11.92GiB, the operating system is Ubuntu16.04 (64-bit), the experimental platform is the Tensorflow 1.10.0 deep learning framework developed by Facebook, and the development language is a python language using Pycharm as python development tool to facilitate better debugging and management of the program.

The experimental data uses domestic flight data from 2019 to 2020. The experiment in this chapter uses the total flight data set. The data set contains 72 flight and meteorological features. The features finally input into the neural network are a three-dimensional matrix.

The structure of the flight delay prediction model based on Att-Conv-LSTM is designed according to Figure 1. The network with different layers in the feature extraction part has the same structure except for the number of filters in the dense block. In this experiment, a random seed is set for the current GPU to ensure consistent training results each time. The experimental hyperparameters mainly include the selection of loss function and optimizer, the setting of learning rate correlation value, etc. The complete hyperparameter configuration of the experiment is shown in Table 2.

### 4.2. Influence of Meteorological Data on Model Performance

This subsection discusses the improvement in model performance brought about by meteorological data in regression models. Table 3 lists the comparison of prediction errors based on the Conv-LSTM network, Beijing Capital Airport fused, and unfused meteorological data.

According to the data in the table, it can be seen that in the regression model, the integration of meteorological data with 1 min precision did not bring about an increase in the sample size, but rather a decrease due to data pre-processing that removed rows with a small number of null values in the meteorological data features. The error after incorporation was reduced by 7.21.

### 4.3. The Effect of Sequence Length on Model Performance

In the regression model, the network used in this paper is more effective for information extraction in the time dimension. Since the sequence length is an important parameter in the recurrent neural network series, this section will discuss the impact of the sequence length on the network results during the serialization process. In this paper, five different step size parameters are tested, and the data set used is the flight and meteorological data of Beijing Capital Airport. The final accuracy is shown in Table 4.

Table 4 lists the change of the accuracy with the increase in sequence length. The comparison shows that when the sequence length is 10, the RMSE reaches the lowest value of 9.82.

**Figure 9:**
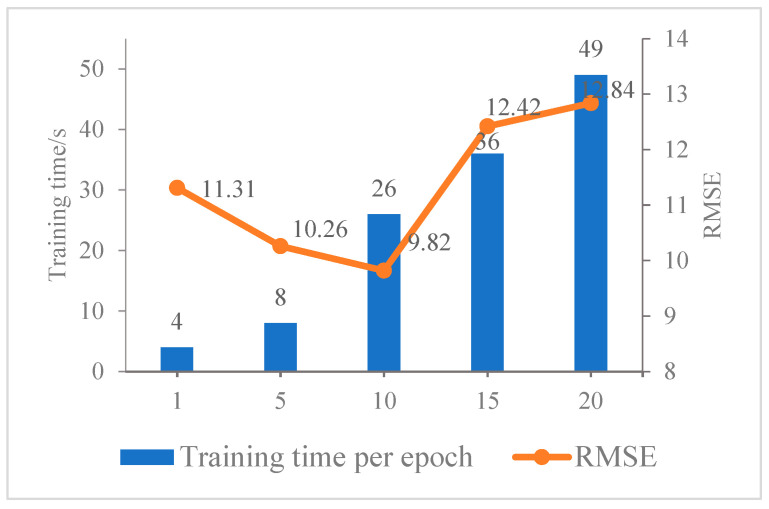
Trend plots of sequence length versus loss and training time.

Figure 9 shows a graph of the model error RMSE versus training time as the sequence length increases. It can be seen from the figure that with the increase in the sequence length, the error of the model shows a downward trend. When the sequence length reaches 10, the error value is the lowest, but it is not that the longer the sequence length, the model performs better. As sequence length increases, the data contains more information, and the prediction results may be more accurate. However, the larger the sequence length is, the more difficult it is to capture the change of flight status in the short term, because there is a short-term temporal correlation between the states of flights, and flight states with too long a time gap have very little or no effect on the state of flights at the current time. It also causes the network to learn irrelevant information which causes data redundancy and increases the prediction error. When it is greater than 10, the model prediction error begins to decrease, indicating that flight delays only exist in a certain length of time series. At the same time, as the sequence length increases, the training time also increases gradually.

The influence of the flight status after a long time on whether the flight at the current moment is delayed has been small or even disappeared. Therefore, the network will learn irrelevant information, resulting in data redundancy and increased errors. In addition, longer time series will consume more training time. Therefore, it is necessary to experiment to choose the appropriate sequence length so that the prediction error is within a small range. From the comparison of several sets of data in the chart, it can be seen that, in the regression model, 10 is a more appropriate time series length value. Therefore, in this paper, the value of the sequence length is 10 as the basic parameter of the subsequent experiments.

### 4.4. Comparative Analysis with Traditional Flight Delay Prediction Methods

In order to verify that the Att-Conv-LSTM method based on deep learning has greater advantages over traditional algorithms in terms of data processing as well as prediction accuracy, the results of different flight delay prediction models are compared separately as shown in Table 5. Several regression models, Linear Regression [11], Decision Tree Regression [12], and Random Forest Regression [12], are trained on the Shijiazhuang Zhengding Airport dataset which is described in detail in Section 2.1.1. Among the above methods, Linear Regression constructs linear functions for input and output values, and Decision Tree Regression and Random Forest Regression are two classical traditional machine learning regression methods. With the same data set, Linear Regression has the largest error RMSE value, while Att-Conv-LSTM has the smallest error RMSE value. The effectiveness of the Att-Conv-LSTM method is verified by comparing it with several regression analysis methods.

### 4.5. Comparative Analysis with Different Time-Series Neural Network Flight Delay Prediction Methods

In this section, the experiment mainly compares the training errors of three different network models from the size of the loss value and discusses the role of model improvement in the iteration of the neural network algorithm.

The loss function is used to estimate the degree of inconsistency between the predicted value f(x) of the model and the real value y. It is a non-negative real-valued function, usually represented by L(y,f(x)). In general, the smaller the loss function, the better the robustness of the model and the better the performance. Table 6 shows the loss values of the Bi-LSTM network, Conv-LSTM network, and Att-Conv-LSTM network model training.

It can be seen from the experimental results in Table 6. Compared with Bi-LSTM, the Conv-LSTM network adds the convolution part, and the error is reduced by an average of 11.41% in the datasets of the four airports; compared with Conv-LSTM, Att-Conv-LSTM network adds an attention mechanism to further extract the time information in the data, and the error is reduced by an average of 10.83%.

**Figure 10:**
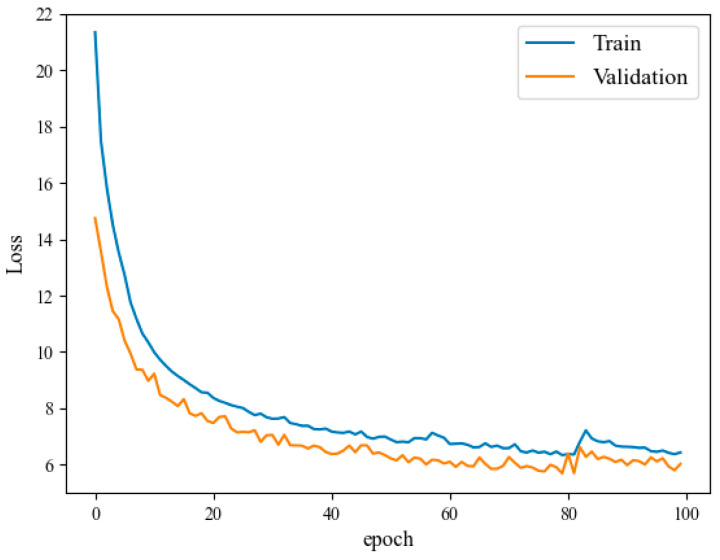
Loss value curve of Shijiazhuang Zhengding Airport in Att-Conv-LSTM model.

Figure 10 shows the decreasing trend of the training loss value based on the Att-Conv-LSTM network under the Shijiazhuang Airport dataset. The horizontal axis represents the number of training rounds, and the vertical axis represents the loss value. The sample size of the training set accounts for 80%, the sample size of the validation set accounts for 20%, and the number of model training rounds is 100 rounds. The sequence length of this experiment is 10, and the other hyperparameter configurations remain the same as in Table 2.

The curves of the training set and the validation set of the four airports in this paper all fit well. Generally, the smaller the loss value, the better the training effect. As can be seen from the above figure, under the Att-Conv-LSTM network model, the loss values of the training set and the validation set decrease gently, and the fitting is good which is in line with the declining law of the general deep learning model of which the RMSE of the validation set is the lowest, reaching 6.81.

### 4.6. Feature Dimension Analysis of Attention Mechanism

In the classification model and regression model, the time dimension is the most important information in the network feature extraction, and the time series length is also an important parameter in the model. The attention mechanism added in the improved model can assign a weight to each step size in each sample. This section visualizes the weight parameters in the two models in order to see more intuitively the effect size of the time series in the network.

**Figure 11:**
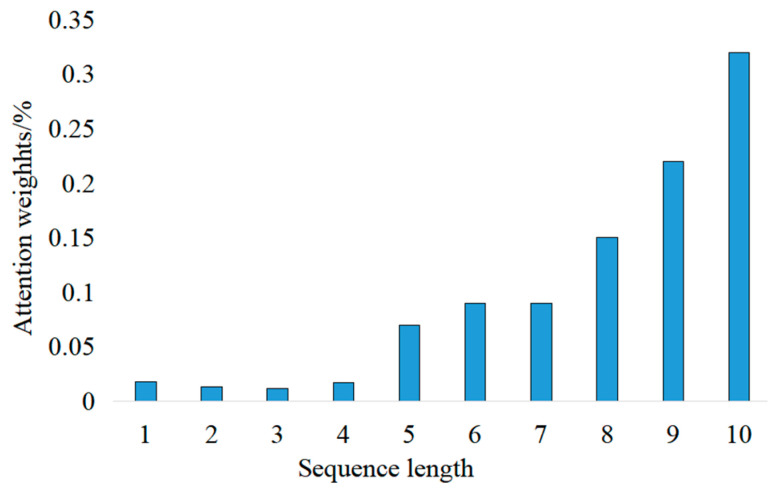
Attention feature dimension map under regression model.

In the experimental analysis in Section 4.3, the sequence length value of 10 is the most suitable choice for the regression model. Similarly, the weight of the attention feature is visualized as shown in Figure 11. It can be seen that the closer to the forecast data to be predicted, the more weighted the bar data vector is in a sample with a stride of 10.

## 5. Conclusions

To give more accurate flight delay prediction results, a flight delay regression prediction model Att-Conv-LSTM is proposed in this paper. The model can predict the specific delay time using spatio-temporal neural network and the attention mechanism module and considering meteorological information. The effectiveness of the model is verified in the dataset of four airports in Beijing, Tianjin, and Hebei. The main conclusions of the paper are as follows:

1. Compared with a single temporal neural network, a higher accuracy can be obtained by simultaneously extracting time series features and spatial features in the data. At the same time, the attention module is added to further improve the learning efficiency of the network.
2. The flight delay status has a short-term time correlation, so the length of the time series in the data is an important parameter in the Att-Conv-LSTM network. If the sequence is too long, it will result in data redundancy and reduce prediction accuracy. For the four domestic airport datasets selected in this paper, when the sequence length is 10, the model can achieve the best performance.
3. Meteorological factors are one of the most important factors that cause flight delays. After adding meteorological data, the dataset becomes more feature attributes, the convolution structure in the algorithm can play a greater role, and the accuracy rate will be improved accordingly.

The follow-up research will focus on feature engineering, how to improve the accuracy of the model by improving the quality of the dataset, and study the realization of the regression model to predict the specific delay time.

## Figures and Tables

**Table 1:** Network structure composition and parameter quantity.

Network Layer	Output Dimension	Parameter Quantity
Reshape	(15, 8, 9, 1)	0
Att-Conv-LSTM	(15, 8, 9, 8)	1472
Batch-Normalization	(15, 8, 9, 8)	32
Att-Conv-LSTM	(15, 8, 9, 16)	9280
Batch-Normalization	(15, 8, 9, 16)	64
Att-Conv-LSTM	(15, 8, 9, 8)	5792
Batch-Normalization	(15, 8, 9, 8)	32
Dense	(1)	8641

**Table 2:** Experimental environment parameters.

Main Parameters	The Parameter Value
Loss function	Cross entropy
Learning rate	0.00001
Optimizer	Adam ($\beta_{1}$ = 0.9, $\beta_{2}$ = 0.999)
Batch normalization	$\xi =10^{-3}$
The number of filters per layer	4/8/4
Convolutional kernel size	(3 × 3)
Sequence length	15
Batchsize	256
Epoch	100

**Table 3:** The effect of meteorological data on errors.

Dataset	Amount of Data	RMSE
Flight data	59,724	17.52
Flight data with meteorological data	58,586	10.31

**Table 4:** Effect of sequence length on prediction error and training time.

Sequence Length	Training Time per Epoch	RMSE
1	4 s	11.31
5	8 s	10.26
10	26 s	9.82
15	36 s	12.42
20	49 s	12.84

**Table 5:** RMSE under traditional flight delay prediction methods.

Model	RMSE
Linear Regression	16.04
Decision Tree Regression	10.03
Random Forest Regression	12.12
Att-Conv-LSTM	6.81

**Table 6:** RMSE under different time-series regression models.

Dataset	Bi-LSTM	Conv-LSTM	Att-Conv-LSTM
Beijing Capital Airport	10.75	9.82	8.97
Beijing Daxing Airport	9.16	7.87	7.23
Tianjin Binhai Airport	10.27	9.12	8.25
Shijiazhuang Zhengding Airport	9.31	8.21	6.81

## Data Availability

Not applicable.
